# Efficacy and Safety of Ivermectin and Hydroxychloroquine in Patients with Severe COVID-19: A Randomized Controlled Trial

**DOI:** 10.3390/idr14020020

**Published:** 2022-03-03

**Authors:** Jose Lenin Beltran Gonzalez, Mario González Gámez, Emanuel Antonio Mendoza Enciso, Ramiro Josue Esparza Maldonado, Daniel Hernández Palacios, Samuel Dueñas Campos, Itzel Ovalle Robles, Mariana Jocelyn Macías Guzmán, Andrea Lucia García Díaz, César Mauricio Gutiérrez Peña, Lucila Martinez Medina, Victor Antonio Monroy Colin, Jose Manuel Arreola Guerra

**Affiliations:** 1 Internal Medicine Department Centenario Hospital Miguel Hidalgo, Aguascalientes 20259, Mexico; dr.jlbg@gmail.com (J.L.B.G.); emmanuel.m.enciso@gmail.com (E.A.M.E.); josue_esparza_@hotmail.com (R.J.E.M.); blue.daniel3@gmail.com (D.H.P.); medintsdc@gmail.com (S.D.C.); itzel.ovrob@gmail.com (I.O.R.); marianamaguz@gmail.com (M.J.M.G.); diazandylucy@gmail.com (A.L.G.D.); mauriciogtz94@gmail.com (C.M.G.P.); 2 Pediatrics Department Centenario Hospital Miguel Hidalgo, Aguascalientes 20259, Mexico; lucymar61@hotmail.com (L.M.M.); vmonroyc@yahoo.com.mx (V.A.M.C.)

**Keywords:** COVID-19, ivermectin, hydroxychloroquine, severe pneumonia

## Abstract

During the first year of the COVID-19 pandemic, unauthorized drugs were widely used. Ivermectin and hydroxychloroquine are drugs that inhibit viral replication in vitro and that have been used in several medical centers. This clinical trial analyzes their efficacy in hospitalized patients with moderate COVID-19. **Methods:** This a controlled, clinical, randomized, double-blind trial that included hospitalized patients with COVID-19-induced pneumonia, without severe respiratory failure. Patients were randomized to one of three groups: Group 1—hydroxychloroquine, 400 mg every 12 h on the first day and, subsequently, 200 mg every 12 h for 4 days; Group 2—ivermectin, 12 mg or 18 mg, according to patient weight; and Group 3—placebo. At inclusion, blood samples for arterial blood gases and biochemical markers were obtained. The primary outcome was established as the length of stay due to patient improvement and the rate of respiratory deterioration or death. **Results:** During the month of August 2020, the admission of patients requiring hospitalization mostly encompassed cases with severe respiratory failure, so we ended the recruitment process and analyzed the data that was available at the time. One hundred and six (106) patients with an average age of 53 yrs (±16.9) were included, with a greater proportion of males (n = 66, 62.2%). Seventy-two percent (72%) (n = 76) had an associated comorbidity. Ninety percent (90%) of patients were discharged due to improvement (n = 96). The average duration of hospitalization was 6 days (IQR, 3–10). No difference in hospitalization duration was found between the treatment groups (Group1: 7 vs. Group 2: 6 vs. Group 3: 5, *p* = 0.43) nor in respiratory deterioration or death (Group 1: 18% vs. Group 2: 22.2% vs. Group 3: 24.3%, *p* = 0.83). **Conclusions:** In non-critical hospitalized patients with COVID-19 pneumonia, neither ivermectin nor hydroxychloroquine decreases the number of in-hospital days, respiratory deterioration, or deaths.

## 1. Introduction

On 30 January 2020, the World Health Organization declared a global health emergency due to SARS-CoV-2 infections (COVID-19) [1]. Since then, the outbreak has spread to all continents and the number of confirmed cases continues to increase. The management of patients that develop symptoms and require hospitalization is mostly supportive.

Chloroquine and hydroxychloroquine belong to the aminoquinoline drug family and are broadly used as a result of their immunomodulatory and potentially antiviral effects and their well-established safety profile^2^. Since the development of the SARS-CoV public health emergency in Southern China in 2003, these drugs have been considered potentially therapeutic [2]. As a consequence, chloroquine and its analog hydroxychloroquine have been proposed as a prophylactic and therapeutic alternative in the management of the different COVID-19 clinical presentations; however, they have not been shown to improve clinical outcomes [3,4,5].

In the absence of specific drugs against COVID-19, potentially active drugs were widely used. One of the most studied is ivermectin, a macrolide obtained from *Streptomyces avermitilis*. The FDA (Food and Drug Administration) authorized the use of this antiparasitic agent in humans in 1998. Significant ivermectin in vitro antiviral effects against SARS-CoV-2 have been recently reported, leading to the development of several clinical studies to determine its efficacy in COVID-19 [6,7].

We aim to evaluate the efficacy and safety of hydroxychloroquine and ivermectin in hospitalized patients with moderate pneumonia secondary to COVID-19.

## 2. Methodology

This is a controlled, randomized, double-blind, clinical trial including patients with pneumonia secondary to SARS-CoV-2 infection and that fulfilled hospitalization criteria. These criteria were defined according to the attending physician in the emergency department and included the following parameters: severity of clinical presentation (determined with the CURB-65 scoring system), need for supplemental oxygen, the presence of comorbidities, and laboratory markers suggesting a poor prognosis (High D-Dimer, Ferritin, Troponin, Creatinine).

The patients included in the study had to fulfill the operational definition of a suspected or confirmed COVID-19 case as well as the pneumonia American Thoracic Society criteria [1,8]. The following patients were considered: (1) positive real-time polymerase chain reaction (RT-PCR) for SARS-CoV-2 by nasal and oropharyngeal swabbing, (2) pneumonia, diagnosed by an X-ray or high-resolution chest computed tomography (CT) scan, with a pattern suggesting involvement due to coronavirus, and (3) recently established hypoxemic respiratory failure or acute clinical deterioration of pre-existing lung or heart disease.

Patients were excluded if they required high oxygen volumes (face mask > 10 L/ min), if they had predictors of a poor response to high-flow oxygen nasal prong therapy, or if they required mechanical ventilation [9]. In the absence of these exclusion criteria, patients were included regardless of other risk factors for poor prognosis.

Patients were classified as high- or low-risk for the development of QT interval prolongation due to hydroxychloroquine according to their electrocardiogram. The QT interval was measured with Bazett’s formula. Patients with an interval of ≥500 ms were randomized to ivermectin or placebo, while those with an interval of <500 ms were randomized to ivermectin, hydroxychloroquine, or placebo. The dose of ivermectin was 12 mg in patients weighing less than 80 kg and 18 mg in those above 80 kg [10].

In the hydroxychloroquine group, patients were administered 400 mg every 12 h on the first day, followed by 200 mg every 12 h for another 4 days. Due to the physical appearance of hydroxychloroquine, calcium citrate was chosen as a placebo and was administered as 2 tablets every 12 h on the first day, followed by one tablet every 12 h for the following 4 days. Blinding was assured with amber-colored vials. Each patient had a vial for the initial dose in order to blind the ivermectin and a second vial for subsequent doses. All patients received two vials, one with the initially prescribed dose and a second one with the indication to take two tablets 12 h after the initial dose followed by one tablet every 12 h until all tablets were finished.

On admission, blood samples were obtained to determine arterial blood gases, a complete blood count, blood chemistry, and prognostic markers such as fibrinogen, D-dimer, ferritin, troponin I, procalcitonin, C-reactive protein, prothrombin, and activated partial thromboplastin times. If available, a high-resolution chest CT scan was also obtained and if not, only a chest X-ray was. The diagnostic probability of pneumonia due to SARS-CoV-2 was established following the COVID-19 Reporting and Data System (CO-RADS) classification [11].

All hospitalized patients received pharmacological thromboprophylaxis with low molecular weight heparin or unfractionated heparin according to local and international guidelines [12,13].

During the last week of June 2020 and based on the RECOVERY trial, we initiated the administration of dexamethasone, 6 mg IV every 24 h, for 10 days or until discharge, in patients requiring oxygen therapy [14].

All included patients were under continuous cardiac monitoring, and protocolized therapy was withdrawn in patients that developed any arrhythmia or acute coronary syndrome.

The efficacy outcomes were defined based on three criteria: total duration of hospitalization, proportion of respiratory deterioration, and death. The safety outcomes, tolerance, and adverse events were also assessed.

Respiratory deterioration was defined as a respiratory frequency above 30 breaths per minute, a required inspired oxygen fraction delivered by face mask or high-flow nasal prongs of 60% or above, a PaO2/Fio2 ratio < 200, or a ROX index at 12 h < 3.85 points [9,15].

Hospital discharge was considered when the patient fulfilled the following criteria: absence of neurologic complications, no fever, hemodynamic stability over at least the previous 72 h, minimal oxygen requirements (nasal prongs at 1–2 L per minute), and the availability of a well-established social support network.

This study was conducted at the *Hospital Centenario Miguel Hidalgo* in the state of Aguascalientes (Mexico), a tertiary care institution for the population lacking social security.

The study protocol was approved by the Ethics Committee of the *Hospital Centenario Miguel Hidalgo* on 15 April 2020, with the assigned number 2020-R-24. It was also included in the *ClinicalTrials* website with the identifier NCT04391127.

## 3. Statistical Analysis

Depending on the measurement level, descriptive statistics were used. The distribution of continuous variables was determined with the Kolmogorov–Smirnov test. Continuous variables with a normal distribution are expressed in means and their standard deviation, while those with an abnormal distribution, as medians and their interquartile ranges. Categorical variables are presented as relative and absolute frequencies. Between-group analysis was evaluated with variance analysis (ANOVA) or the Kruskal–Wallis test, depending on the distribution. Dichotomous or ordinal variables were analyzed by chi-square or Fisher’s exact test, as needed. Survival analysis was performed for the outcomes of death or respiratory deterioration, with Kaplan–Meier curves, and between-group comparisons were obtained with the Log-rank test. A *p* value below 0.05 was considered significant. Microsoft Excel 2013 and STATA version 11.1 software were used for analysis.

Considering a mean hospitalization stay of 20 to 30 days and standard deviation of 7 days, if we want a reduction of 4 days of hospitalization, taking into account the mean differences formula, we calculate 47 patients per group of treatment.

## 4. Results

During the month of August 2020, we observed a very significant decrease in the number of potential candidates that could be included in the study, since practically all hospital admissions required therapy with high oxygen concentrations or invasive mechanical ventilation. Based on the Ethics Committee’s recommendations, we decided to end recruitment and conduct an analysis with the data obtained as of 15 August 2020.

At the time of analysis, 108 patients had been recruited, two of which were eliminated because they were transferred to another hospital. The patients’ average age was 53 years (±16.9), and there was a higher proportion of males (n = 66, 62.2%). Median time of symptoms onset was 7 days before of admission, with no differences between groups. Comorbidities were present in 72% of cases (n = 76). Type 2 diabetes mellitus and systemic arterial hypertension were the most common comorbidities (33.9 and 32.1%, respectively). Mean body weight was 82.3 kg (±19.6) with a BMI of 29.6. (±6.6) (Table 1).

A high-resolution chest CT scan was obtained in 93 patients. On admission, 69 patients had typical infiltrates characteristic of SARS-CoV-2 infection pneumonia, defined by CO-RADS 5, while 24 patients had an image with a low probability of SARS-CoV-2 infection according to that scoring system, but they had a diagnostically compatible clinical presentation and/or a positive RT-PCR test for SARS-CoV-2. (Table 2).

The arterial oxygen pressure (PaO2) and the inspired oxygen fraction ratio (PaO_2_/FiO_2_) was greater than 200 mmHg in 64 patients; 42 patients had severe respiratory deterioration (Table 3).

The rate of patients with cutoff points of poor prognosis in severity scales was as follows: in 77%, the Neutrophil/Lymphocyte index was ≥3; SOFA ≥ 2 in 93%; APACHE ≥ 8 points in 96%; and CURB-65 ≥ 2 points in 34.9%. The score in severity scales was not different between groups (Table 4).

During hospitalization, 52.3% of patients received antibiotics, 92.2% were on thromboprophylaxis, and 55.7% received dexamethasone as ancillary therapy (Table 5).

**Figure 1:**
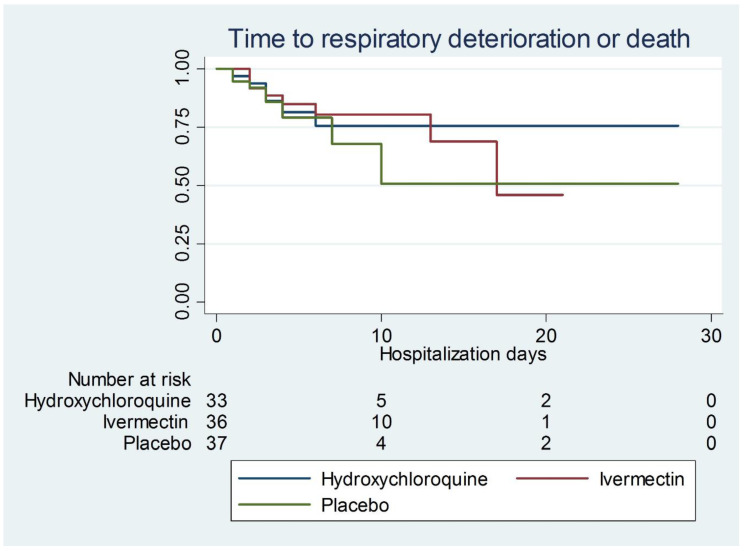
Time to compound outcome of death or respiratory deterioration (*p* = 0.44).

The average duration of hospitalization was 6 days (IQR 3–10). Ninety percent (90%) of patients were discharged after clinical improvement (n = 96). Twenty-three (23) patients developed respiratory deterioration, thirteen (12.2%) of whom died during their hospital stay, all as a result of respiratory failure, and three with sepsis due to bacterial coinfection. No differences in outcome were detected between the treatment groups (Table 6). The time until death or respiratory deterioration was also not different between groups (Figure 1).

During hospital stay there was no thrombotic complications, and no arrhythmias developed in patients on hydroxychloroquine. Only three patients developed septic shock, one in each treatment arm. No patient presented intolerance or adverse events related to the administration of hydroxychloroquine or ivermectin.

## 5. Discussion

In this study of non-critically ill patients with pneumonia secondary to COVID-19 and fulfilling hospitalization criteria, treatment with hydroxychloroquine or ivermectin was not superior to placebo, neither in terms of hospitalization duration nor in progression to severe respiratory failure or death.

In the first weeks of the COVID-19 pandemic, there was in vitro evidence on the efficacy of hydroxychloroquine as well as case series and non-controlled comparative studies supporting its use. This led various medical centers and health systems to recommend it based on compassionate use. This strategy fostered panic purchases and prescriptions leading to drug shortages for patients with well-established indications. As months went by, its inefficacy was suspected and reports from the SOLIDARITY study finally proved its therapeutic futility in decreasing mortality, and patient recruitment was stopped [16]. Other analysis further confirms that study’s findings [4,5].

Although adverse events have been reported with the use of hydroxychloroquine, particularly QT interval prolongation, none of our patients developed cardiovascular complications associated with hydroxychloroquine use; perhaps electrocardiographic screening may have contributed to the avoidance of these complications.

As with hydroxychloroquine, ivermectin was proposed in the early phases of the pandemic as a treatment and even prophylaxis of SARS-CoV-2 infection. It has mainly been used in Latin America as compassionate treatment with no evidence supporting its efficacy in COVID-19; some health ministries have even modified their treatment policies recommending its generalized use [17].

As in the case of hydroxychloroquine, evidence in favor of ivermectin use was the result of in vitro studies and non-controlled comparative trials [18]. A possible explanation for those findings is the fact that the in vitro efficacy of ivermectin in decreasing the viral load of SARS-CoV-2, is clinically translated in much greater ivermectin doses in vivo, an unplausible proposal in the clinical setting [19]. To our knowledge, at the time of the study’s conclusion (September 2020), it was the first clinical trial comparing ivermectin with placebo [20].

The characteristics of patients enrolled in our study differ from those in series published in China and some European countries early on since our population has a greater incidence of overweight and obesity.

The patients included have a greater load of comorbid disease, thus impacting clinical results. As in the initial case series, systemic arterial hypertension and type 2 diabetes mellitus were the main entities compromising the patients’ course and fostering their development of respiratory insufficiency and an increase in adverse clinical outcomes.

The main strength of this clinical trial is the fact that it represents the response of a referral hospital in the management of patients with COVID-19. In a controlled manner, patients were offered two therapeutic alternatives that at the beginning of the pandemic appeared to be potentially effective. Although the patient number is not sufficient to reach categorical conclusions, the study’s design certainly suggests that both drugs are ineffective. We hope it will contribute to meta-analyses that may yield more robust conclusions.

The study’s main weakness is the limited number of patients per group and low statistical power shown in important outcomes such as death (25%); also, among the pre-established outcomes, we were unable to determine whether the SARS-CoV-2 PCR tests became negative, due to the lack of reactants and the minimal usefulness of proving its negativity from a clinical-practical viewpoint.

## 6. Conclusions

In non-critically ill, hospitalized patients with pneumonia secondary to COVID-19, the use of hydroxychloroquine or ivermectin did not decrease significantly the number of hospitalization days, respiratory deterioration, or deaths.

## Figures and Tables

**Table 1:** Population characteristics.

Variables	Entire Group (n = 106)	Hydroxychloroquine (n = 33)	Ivermectin (n = 36)	Placebo (n = 37)	*p*
**Age, m (±S)**	53.8 (16.9)	48.9 (15.3)	56 (16.5)	53.8 (16.9)	0.15
**Males, n (%)**	66 (62.2)	22 (66.6)	21 (58.3)	23 (62.1)	0.77
**Diabetes Mellitus, n (%)**	36 (33.9)	11 33.3)	9 (25)	16 (43.2)	0.25
**SAH, n (%)**	34 (32.1)	8 (24.2)	12 (33.3)	14 (37.8)	0.47
**CKD, n (%)**	5 (4.7)	2 (6.1)	2 (5.5)	1 (2.7)	0.73
**COPD, n (%)**	7 (6.6)	1 (3)	2 (5.5)	4 (10.8)	0.50
**Healthy, n (%)**	30 (28.3)	13 (39.3)	10 (27.7)	7 (18.9)	0.17
**Weight, m (±S)**	82.3(19.6)	84.8 (20.8)	80 (19.7)	82 (18.2)	0.66
**BMI, m (±S)**	29.6 (6.6)	30.3 (6.3)	29.2 (7)	29.4 (6.6)	0.55
**D symptoms onset, med (±IQR)**	7 (4–10)	7 (5–12)	6 (4–10)	7 (5–10)	0.84
**D of +RT-PCR, med (IQR)**	+1 (0–2)	+1 (−1–2)	+1 (0–2)	+1 (1–2)	0.41

S: standard deviation, med: Median, IQR: interquartile range SAH: Systemic Arterial Hypertension, CKD: Chronic Kidney Disease, COPD: Chronic Obstructive Pulmonary Disease, BMI: Body Mass Index. +RT-PCR: positive polymerase chain reaction for SARS-CoV-2. D: Days.

**Table 2:** Computed Tomography Characteristics.

Variables, n (%)	Entire Group (n = 93)	Hydroxychloroquine (n = 28)	Ivermectin (n = 30)	Placebo (n = 35)
**CO-RADS 2**	5 (5.3)	0	3 (10)	2 (5.2)
**CO-RADS 3**	15 (16.1)	4 (14.2)	7 (23.3)	4 (11.4)
**CO-RADS 4**	19 (20.4)	7 (25)	4 (13.3)	8 (22.8)
**CO-RADS 5**	50 (53.7)	17 (25)	13 (43.3)	20 (57.1)
**CO-RADS 6**	4 (4.3)	0	3 (10)	1 (2.8)

CO-RADS: COVID-19 Reporting and Data System classification.

**Table 3:** Biochemical and gasometric markers.

Variables	Entire Group (n = 106)	Hydroxychloroquine (n = 33)	Ivermectin (n = 36)	Placebo (n = 37)	*p*
**SatO_2_, m (±S)**	84 (7)	86 (9)	83 (8)	83 (8)	0.27
**PaO_2_/FiO_2_, m (±S)**	223 (103)	224 (116)	245 (107)	201 (82)	0.17
**<100, n (%)**	11 (10.3)	5 (15.1)	2 (5.5)	4 (10.8)	0.44
**100–200, n (%)**	31 (29.2)	8 (24.2)	9 (25)	14 (37.8)	0.36
**>200–300, n (%)**	48 (45.2)	17 (51.5)	16 (44.4)	15 (40.5)	0.64
**>300, n (%)**	16 (15.1)	3 (9.1)	9 (25)	4 (10.8)	0.14
**PCO_2_, m (±S)**	29 (7.5)	26.3 (7.9)	30.4 (7.2)	30.3 (6.8)	0.09
**Lactate, m (±S)**	1.41 (0.6)	1.45 (0.7)	1.2 (0.6)	1.5 (0.6)	0.31
**Hb, m (±S)**	13.5 (2.9)	13.6 (3)	13.1 (3)	13.7 (2.8)	0.63
**Leukocytes, m (±S)**	10 (4.4)	9.6 (3.5)	10.3(5.1)	10.1 (4.5)	0.92
**Neutrophils, m (±S)**	8 (4.1)	7.7 (3.4)	8.4 (4.8)	7.8 (4.1)	0.82
**Lymphocytes, m (±S)**	1.3 (0.6)	1.3 (0.54)	1.2 (0.6)	1.4 (0.7)	0.4
**Platelets, m (±S)**	254 (104)	250 (91)	261 (100)	251 (119)	0.9
**Creatinine, m (±S)**	1.48 (2.8)	0.95 (0.7)	1.6 (3.3)	1.8 (3.4)	0.81
**LDH, m (±S)**	394 (158)	394 (189)	369 (123)	394 (158)	0.19
**D-Dimer, m (±S)**	1618 (1442)	1593 (1845)	1872 (1137)	1380 (1268)	0.01
**Fibrinogen, m (±S)**	441 (247)	439 (249)	473 (250)	413 (244)	0.53
**CRP, m (±S)**	117 (136)	171 (122)	187 (126)	172 (158)	0.67
**Ferritin, m (±S)**	721 (927)	669 (667)	902 (1125)	592 (910)	0.35
**D Bilirubin, m (±S)**	0.64 (1.6)	0.91 (2.9)	0.53 (0.5)	0.5 (0.6)	0.56
**Troponin, m (±S)**	0.05 (0.2)	0.1 (0.3)	0.03 (0.08)	0.02 (0.02)	0.18

O2Sat: oxygen saturation, PaO2/FiO2: index of oxygen arterial pressure/inspired oxygen fraction, PCO2: carbon dioxide pressure, Hb: hemoglobin, LDH: Lactic Dehydrogenase, CRP: C-reactive protein.

**Table 4:** Markers and prognostic score systems.

Variables	Entire Group (n = 106)	Hydroxychloroquine (n = 33)	Ivermectin (n = 36)	Placebo (n = 37)	*p*
**N/L Index ≥ 3, n (%)**	89 (77.7)	30 (90.9)	31 (86.1)	28 (77.7)	0.34
**SOFA, m (S)**	3.3 (1.6)	3.1 (1.5)	2.9 (1.5)	3.8 (1.7)	0.05
**SOFA < 2, n (%)**	7 (6.6)	2 (6.1)	4 (11.1)	1(2.7)	0.33
**2–3, n (%)**	61 (57.6)	21 (63.6)	22 (61.1)	18 (48.6)	0.38
**≥4, n (%)**	38 (35.8)	10 (30.3)	10 (27.7)	18 (48.6)	0.14
**APACHE II, m (±S)**	11.2 (4)	11.1 (3.8)	10.6 (3.7)	11.9 (4.4)	0.38
**8–15, n (%)**	67 (63.2)	22 (66.6)	23 (63.8)	22 (59.4)	0.14
**>15, n (%)**	23 (21.2)	7 (21.2)	5 (13.8)	11 (21.7)	0.25
**CURB65, m (±S)**	1.08 (1.03)	0.9 (0.9)	1.1 (1)	1.1 (1.1)	0.77
**≥2, n (%)**	37 (34.9)	11 (33.3)	14 (38.8)	12 (32.4)	0.82

N/L: Neutrophil/Lymphocyte, SOFA: Sequential Organ Failure Assessment Score, APACHE: Acute Physiology And Chronic Health Evaluation II, CURB65: confusion, uremia, respiratory rate, BP, age ≥ 65 years.

**Table 5:** Ancillary treatments.

Variables, n (%)	Entire Group (n = 106)	Hydroxychloroquine (n = 33)	Ivermectin (n = 36)	Placebo (n = 37)	*p*
**Antibiotic**	56 (52.8)	15 (45.4)	23 (63.8)	18 (52.8)	0.25
**Cephalosporines**	35 (33)	11 (33)	15 (41.6)	9 (24.3)	0.28
**Macrolides**	17 (16)	5 (15.1)	7 (19.4)	5 (15.5)	0.77
**Quinolones**	10 (9.4)	2 (6)	3 (8.3)	5 (13.5)	0.54
**Piperacillin Tazobactam**	5 (4.7)	0	3 (8.3)	2 (5.4)	0.25
**Carbapenems**	10 (9.4)	2 (6)	6 (16.6)	2 (5.4)	0.18
**Thromboprophylaxis**	101 (92.2)	30 (90.9)	36 (100)	35 (94.5)	0.19
**Steroids**	61 (57.5)	21 (63.6)	21 (58.3)	19 (51.3)	0.6

**Table 6:** Outcomes. IQR: interquartile range.

Outcome	Entire Group (n = 106)	Hydroxychloroquine (n = 33)	Ivermectin (n = 36)	Placebo (n = 37)	*p* Value
**Duration of hospitalization, med (IQR)**	6 (3–10)	7 (3–9)	6 (4–11)	5 (4–7)	0.43
**Hospital discharge, n (%)**	96 (90.5)	30 (90.9)	32 (88.8)	34 (91.8)	0.91
**Discharge without respiratory deterioration or death, n (%)**	80 (75.4)	26 (78.7)	27 (75)	27 (72.9)	0.85
**Respiratory deterioration or death, n (%)**	23 (21.7)	6 (18.1)	8 (22.2)	9 (24.3)	0.83
**Death, n (%)**	13 (12.2)	2 (6)	5 (13.8)	6 (16.2)	0.42

## Data Availability

To obtain the database or any specific data, please contact the corresponding author.
